# *Helicobacter pylori* infection altered gastric microbiota in patients with chronic gastritis

**DOI:** 10.3389/fcimb.2023.1221433

**Published:** 2023-08-17

**Authors:** Zhaolai Hua, Le Xu, Jiahui Zhu, Ling Xiao, Bin Lu, Jianping Wu, Zhenfeng Wu, Qihai Zhou, Junfeng Zhang

**Affiliations:** ^1^ Guangxi Key Laboratory of Rare and Endangered Animal Ecology, Guangxi Normal University, Guilin, China; ^2^ Institute of Tumor Prevention and Control, People’s Hospital of Yangzhong City, Yangzhong, China; ^3^ School of Medicine and Holistic Integrative Medicine, Nanjing University of Chinese Medicine, Nanjing, China; ^4^ Department of Oncology, People’s Hospital of Yangzhong City, Yangzhong, China; ^5^ Department of Surgical Oncology, Jiangsu Province Hospital of Chinese Medicine, Affiliated Hospital of Nanjing University of Chinese Medicine, Nanjing, China

**Keywords:** *Helicobacter pylori*, chronic gastritis, gastric mucosa, microbiota, symbiotic relationship

## Abstract

**Objective:**

The present study aims to investigate the effect of *Helicobacter pylori* (Hp) infection on gastric mucosal microbiota in patients with chronic gastritis.

**Methods:**

Here recruited a population of 193 patients with both chronic gastritis and positive rapid urease, including 124 patients with chronic atrophic gastritis (CAG) and 69 patients with chronic non-atrophic gastritis (nCAG). Immunoblotting was used to detect four serum Hp antibodies (UreA, UreB, VacA and CagA) to determine the types of virulent Hp-I and avirulent Hp-II infections. Gastric microbiota was profiled by 16S rRNA gene V3-V4 region, and R software was used to present the relationship between the microbial characteristics and the type of Hp infection.

**Results:**

In the stomach of patients with Hp-positive gastritis, the dominant gastric bacterial genera included *Ralstonia* (23.94%), *Helicobacter* (20.28%), *Pseudonocardia* (9.99%), *Mesorhizobium* (9.21%), *Bradyrhizobium* (5.05%), and *Labrys* (4.75%). The proportion of Hp-I infection was significantly higher in CAG patients (91.1%) than in nCAG patients (71.0%) (*P* < 0.001). The gastric microbiota richness index (observed OTUs, Chao) was significantly lower in CAG patients than in nCAG patients (*P <*0.05). Compared with avirulent Hp-II infection, virulent Hp-I infection significantly decreased the Shannon index in CAG patients (*P <*0.05). In nCAG patients, Hp-I infected patients had lower abundances of several dominant gastric bacteria (*Aliidiomarina*, *Reyranella*, *Halomonas*, *Pseudomonas*, *Acidovorax*) than Hp-II infected patients. Meanwhile, in CAG patients, Hp-I infected patients occupied lower abundances of several dominant oral bacteria (*Neisseria*, *Staphylococcus* and *Haemophilus*) than Hp-II infected patients. In addition, bile reflux significantly promoted the colonization of dominant oral microbiota (*Veillonella*, *Prevotella 7* and *Rothia*) in the stomach of CAG patients. There was no significant symbiotic relationship between *Helicobacter* bacteria and non-*Helicobacter* bacteria in the stomach of nCAG patients, while *Helicobacter* bacteria distinctly linked with the non-*Helicobacter* bacteria (*Pseudolabrys*, *Ralstonia*, *Bradyrhizobium*, *Mesorhizobium* and *Variovorax*) in CAG patients.

**Conclusions:**

Virulent Hp infection alters the gastric microbiota, reduces microbial diversity, and enhances the symbiotic relationship between the *Helicobacter* bacteria and non-*Helicobacter* bacteria in patients with chronic gastritis. The data provides new evidence for treating Hp infection by improving the gastric microbiota.

## 1 Introduction

Chronic gastritis is a common disease of the gastrointestinal tract with prolonged and slow progression, and is classified into chronic non-atrophic gastritis (nCAG) and chronic atrophic gastritis (CAG) according to the presence of mucosal atrophy under gastroscopy and pathology. The prevalence of CAG increases with age, and differs in national region. Age is positively correlated with the incidence of gastric cancer (GC), and aging plays a key role in the stability of standardized GC incidence and results in the increasing GC cases in China (Xia et al., 2022). CAG is a typical precancerous state of stomach, its clinical transformation features go beyond the strict field of gastroenterology and manifest a variety of complex gastric and extragastric symptoms/signs, and the optimal treatment remains to be determined (Lahner et al., 2019). Therefore, revealing the biological mechanisms of CAG development could provide a key strategy for the prevention and treatment of GC.

*Helicobacter pylori* (Hp) infection is the main pathogen that induces chronic active gastritis, and about 4.4 billion people worldwide are infected with Hp. Most Hp bacteria carry virulent vacuolating cytotoxin (VacA) and/or cytotoxin-associated gene A product (CagA) (Sharndama and Mba, 2022), which significantly enhance the GC risk, and clinical detection of Hp virulence genes has been an important strategy to predict the GC risk (Idowu et al., 2019). Early studies had shown that VacA can result in peptic ulcers (Atherton et al., 1995), and CagA-positive Hp strains have a higher risk of peptic ulcers and gastric cancer than CagA-negative Hp strains (Blaser et al., 1995). Gao et al. (2009) investigated the correlation between 15 kinds of Hp protein antibodies in serum and the GC risk in a German population, and found that the risk of GC in CagA-positive patients was 5.63 times higher than that in CagA-negative patients, and CagA and GroEL seropositivity were independent factors of the GC risk. Similarly, Inagaki et al. (2017) also found that VacA genotyping could predict Hp virulence and GC prognosis in the Japanese population. The latest meta-analysis with 3209 GC cases and 6964 controls showed that serum positive anti-CagA and anti-VacA distinctly increased the risk of non-cardia GC, and the odds ratios were 3.22 and 2.05, respectively (El Hafa et al., 2022). These results suggest that CagA and/or VacA-expressive Hp infection play a key role in promoting the evolution of chronic gastritis to CAG/GC.

To facilitate clinical management of Hp infection, serum anti-CagA and/or anti-VacA positive was defined as virulent Hp-I infection, while neither anti-CagA nor anti-VacA positive was defined as avirulent Hp-II infection. Liu et al. (2021) found that Hp-I infection was positively correlated with gastric mucosal inflammatory activity in the patients with chronic gastritis, and the Hp-I infected patients with had higher levels of serum G-17, PG I, PG II and lower PG I/II ratio than the Hp-II infected patients. Similarly, Yuan et al. (2020) found that Hp-I infection linked with higher serum PG II and lower PG I/PG II ratio in patients with nCAG, non-atrophic gastritis with erosion, and CAG. These results suggest that Hp-I infection is the key driving force in the progression of chronic gastritis to gastric precancerous lesions, and gastric carcinoma.

With the development of culture-independent high-throughput microbiota sequencing, more and more evidences have presented the potential role of non-Hp microbiota in the development of gastric cancer (Gunathilake et al., 2021). Knippel et al. (2021) held that oral microbiota linked with GC risk, for example, elevated abundance of oral *Lactobacillus* promoted the GC risk, and co-infection of Hp/EBV enhanced the development of Hp-related GC. Shen et al. (2022) found that urease-positive *Staphylococcus epidermidis* and *S. salivarius* co-infection with Hp did not affect the colonization of Hp in the stomach of mice, but chronic gastritis caused by Hp/*S. salivarius* coinfection was more severe than Hp alone or Hp/*S. epidermidis* coinfection, suggesting that urease-positive non-Hp bacteria may play a role in the severity of Hp-induced gastric carcinoma. With rapid spread of clarithromycin-resistant Hp infection, the use of probiotics to ameliorate antibiotic side effects had received increasing attention (Sousa et al., 2022), especially in pediatric infections, where probiotics could alter gastric pH value and reduce the adhesion and colonization of Hp in the gastric mucosa (Meliţ et al., 2022). Though most probiotic treatment strategies were effective for Hp eradication, there were still great challenges in the efficiency and tolerability of Hp eradication in triple therapy (Wang et al., 2017). The clinical data implied that gastric non-Hp microbiota also involved in the occurrence of gastritis and gastric carcinogenesis. Therefore, elucidating the symbiotic relationship between Hp infection and gastric microbiota will provide a new strategy for the management of Hp infection via probiotics intervention to restore the microbial balance in the stomach.

To explore the effect of Hp-infection typing on the symbiosis of gastric mucosal microbiota, this study recruited 193 gastritis patients with positive rapid urease test (RUT), four serum antibodies (anti-UreA, anti-UreB, anti-CagA, anti-VacA) were detected to type Hp-infection using immunoblotting dipstick test, gastric microbiota was profiled using 16S rRNA gene high-throughout sequencing. The present study focused on the relationship between Hp-infection typing and gastric microbiota in CAG patients, and found that *Helicobacter* bacteria had a strong symbiotic relationship with the gastric microbiota of CAG patients, but had a scarcely symbiotic relationship with the gastric microbiota of nCAG patients. The results brought new insight into understanding the effect of Hp-infection on chronic gastritis.

## 2 Materials and methods

### 2.1 Study design and ethics

From April 2018 to January 2020, among the endoscopic screening population of upper gastrointestinal cancer in Yangzhong City, 193 individuals diagnosed as chronic gastritis and RUT-positive were brought into this study. According to the consensus opinion on chronic gastritis in China formulated by the Digestive Diseases Branch of the Chinese Medical Association (Shanghai, 2017) (Fang, 2023), “Atrophy and/or intestinal metaplasia” in pathological diagnosis was defined as CAG, and others were defined as nCAG. There were 124 CAG patients and 69 nCAG patients in the 193 individuals, and the clinical data were collected using a unified questionnaire. Exclusion criteria: 1) patients with a history of major organic lesions, such as stroke, malignancy, organ transplantation, gastrointestinal surgery, etc.; 2) history of antibiotics, probiotics, proton pump inhibition and other drugs in the last month; 3) age below 40 or above 70 because most asymptomatic patients screened out in the range of 40 - 70 years old; 4) neoplasia, cancer and other suspected gastric mucosal cancer in pathological diagnosis; 5) residence in Yangzhong area for less than 5 years; 6) unwillingness to cooperate. The study was approved by the Ethics Committee of People’s Hospital of Yangzhong City (No.2018-039) and the patients gave informed consent.

### 2.2 Gastric mucosa sampling and microbiota profiling

Gastric sinus mucosal tissues were clamped during early morning fasting and electronic gastroscopy, quickly frozen in liquid nitrogen and stored at -80°C. Gastric microbiota were profiled according to the previous literature (Xu et al., 2023). Total DNA of gastric mucosa was extracted by QIAamp DNA Mini Kit (Qiagen, Valencia, CA, USA), and the quality of DNA was detected by 1% agarose gel electrophoresis. PCR amplification was performed using universal primers in V3-V4 region of 16S rRNA gene, and Miseq PE library was prepared for high-throughput sequencing. QIIME (Version 1.17) was used to optimize sequencing data, and the operational taxonomic unit (OTU) was established in UP-ARSE35 software according to 97% similarity, and taxonomic annotations of microbiota species were obtained from the RDP (Ribosomal Database Project II database).

### 2.3 Serum Hp antibodies immunoblotting assay and Hp-infection typing

Fasting venous blood 2–3 ml with a procoagulant tube was collected, the serum was separated by 3000 rpm centrifugation for 10min. According to the previous literature (Liu et al., 2021), Hp antibody typing classification assay kit (western blotting) (Shenzhen Blot Biological Products Co., Ltd., No. 201905016) was used to detect Hp-related IgG against four antigens including CagA (116 kD), VacA (95 kD, 91 kD), UreB (66 kD) and UreA (30 kD). The experimental process was briefly described as following: 1) Place the blotting membrane strips into the assay tank, add 1000 μL dilution solution and 10 μL serum, and shake (40 times/min) for 30 minutes at room temperature (22-23°C); 2) Wash the strips with 1000 μL washing buffer for 4 times, add 500 μL working solution and 10 μL enzyme-linked antibodies, and shake (40 times/min) for 30 minutes at room temperature (22-23°C); 3) Wash the strips with 1000 μL washing buffer for 3 times, add 500 μL chromogenic reagent solution, stand for 5 mins, and terminate the reaction by washing 3 times with 1000 μL distilled water.

According to Hp antibody typing classification, Hp infection was divided into Hp-I infection and Hp-II infection. If the bands of CagA and/or VacA were positive, it could be diagnosed as Hp-I infection; if the bands of both CagA and VacA were negative, but the bands of urease A and/or urease B were positive, it could be diagnosed as Hp-II infection; if all of the four bands were negative, it could be diagnosed as non-Hp infection. The band of quality control should appear normally, otherwise, the test should be invalid.

### 2.4 Statistical analysis

SPSS software (Version 25.0, SPSS, inc.) was used for clinical parameter analysis. Visual Genomics software (Release 1, Shanghai Infinity Biotechnology Co., Ltd.) was conducted for alpha diversity and community structure analysis. Spearman correlation analysis was used to construct the symbiotic relationship of the microbiota, and R software (Version 2022.02.0-443, https://www.r-project.org/) was used for volcano plot, Spearman correlation analysis, and graphical representation. Two-tailed *P* < 0.05 indicated significant statistical significance.

## 3 Results

### 3.1 General clinical characteristics

The present 193 individuals included 124 CAG patients and 69 nCAG patients, who were well matched by the gender, body mass index (BMI), and four lifestyle habits (eating fried foods, eating fresh fruits, smoking and drinking) (*P* > 0.05). However, the CAG patients were significantly elder (*P* < 0.05), and had higher proportion of bile reflux and inflammatory activity than nCAG patients (*P* < 0.01). It is worth noting that the CAG patients had a higher proportion of Hp-I infection than nCAG patients (*P* < 0.001). Additionally, there were statistically significant differences in the distribution of eating speed and salt preference (*P* < 0.05) (**Table S1**).

### 3.2 Hp-infection typing altered microbial diversity of gastric microbiota

**Figure 1:**
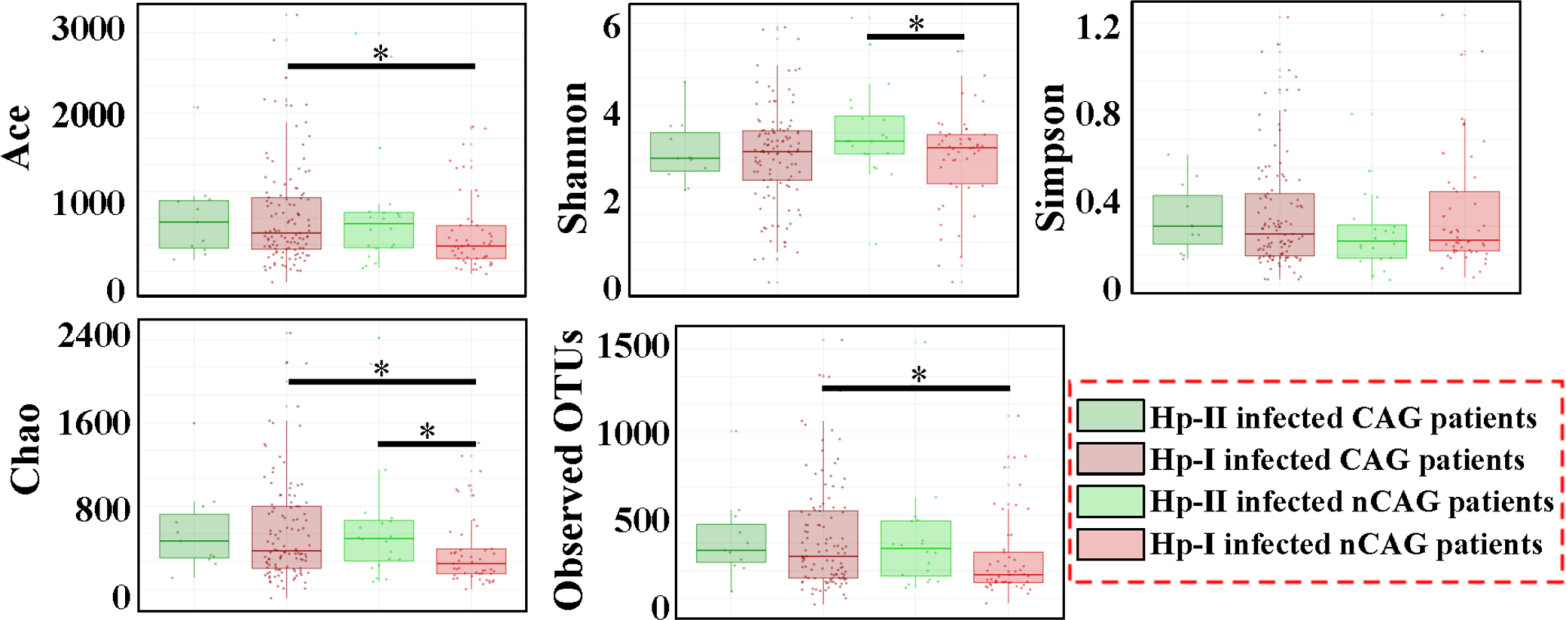
Effect of Hp-infection typing on the alpha diversity of gastric microbiota in patients with chronic gastritis. Non-parametric Mann-Whitney *U* test was used to conducted the different analysis, and the asterisk (*) indicated *P* < 0.05.

Alpha diversity showed that the richness indices (Ace, Chao and Observed OUTs) of gastric microbiota were significantly higher in CAG patients than in nCAG patients (*P* < 0.05) in the patients with Hp-I infection; however, among the patients with Hp-II infection, no differences of the richness indices were observed between CAG patients and nCAG patients (*P* > 0.05). Meanwhile, no significant difference of Shannon was observed between CAG patients and nCAG patients (*P* > 0.05) (**Figure 1**). Interestingly, among nCAG patients, the Hp-I infected patients had significantly lower Chao and Shannon indices than the Hp-II infected patients (*P* < 0.05). The results suggested that Hp-I infection significantly decreased the richness and diversity of gastric microbiota in nCAG patients, while significantly increased the richness of gastric microbiota in CAG patients.

### 3.3 Hp-infection altered the community of gastric microbiota

**Figure 2:**
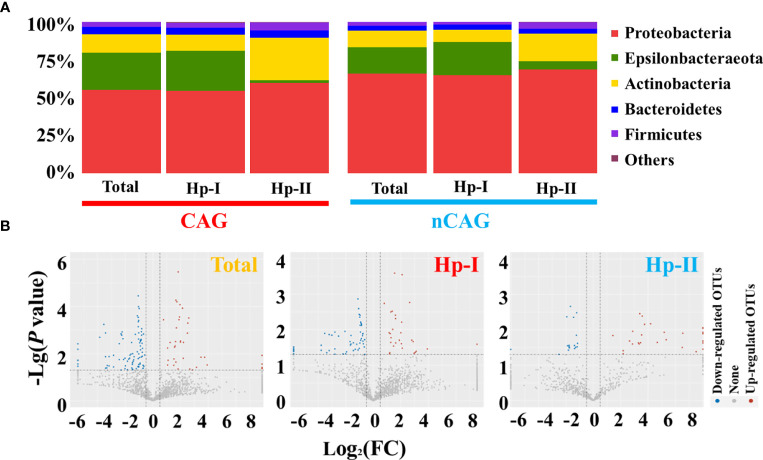
Effect of Hp-infection typing on gastric microbial community in patients with chronic gastritis. **(A)** The stacked bar chart presented the community of gastric microbiota in total population, Hp-I infected patients, and Hp-II infected patients at phylum level. **(B)** Volcano plot showed the different bacteria between nCAG and CAG patients at OTU level.

Here investigated the effect of Hp-infection typing on the gastric microbiota in patients with chronic gastritis (**Figure 2** **)**. At the phylum level, the dominant gastric phyla in gastritis patients included Proteobacteria, Epsilonbacteraeota, Actinobacteria, Bacteroidetes, and Firmicutes, especially Proteobacteria was the most prevalent phylum (relative abundance > 50%) (**Figure 2A**). Among dominant phyla, non-parametric analysis showed that Hp-I infected patients had a higher Epsilonbacteraeota and lower Actinobacteria than Hp-II infected patients (*P* < 0.05). The 17 prevalent bacterial genera (prevalent rate 100%) included *Ralstonia* (23.94%), *Helicobacter* (20.28%), *Pseudonocardia* (9.99%), *Mesorhizobium* (9.21%), *Bradyrhizobium* (5.05%), *Labrys* (4.75%), *Hydrobacter* (3.56%), *Variovorax* (2.63%), *Pseudolabrys* (2.25%), *Reyranella* (1.51%), *Acidovorax* (1.11%), *Methylobacterium* (1.05%), *Aliidiomarina* (0.83%), *Nesterenkonia* (0.68%), *Pseudomonas* (0.62%), *Cupriavidus* (0.43%) and *Pelomonas* (0.25%). At OUT level, different analysis showed that the Hp-infection typing exerted significant effect on the gastric microbial community in patients with chronic gastritis (**Figure 2B**). After calculating the fold of change (FC) in each OUT between CAG and nCAG patients, the different OUTs were collected based on Log_2_(FC) greater than 0.5 or less than -0.5 with a correction of *P* < 0.05. The present study observed 40 significantly up-regulated OTUs and 98 significantly down-regulated OTUs in the gastric mucosa of CAG patients. Among Hp-I infected patients, 29 up-regulated OTUs and 81 down-regulated OTUs were observed in the stomach of CAG patients compared with nCAG patients. Among Hp-II infected patients, 29 up-regulated OTUs and 17 down-regulated OTUs were collected in the stomach of CAG patients compared with nCAG patients.

**Figure 3:**
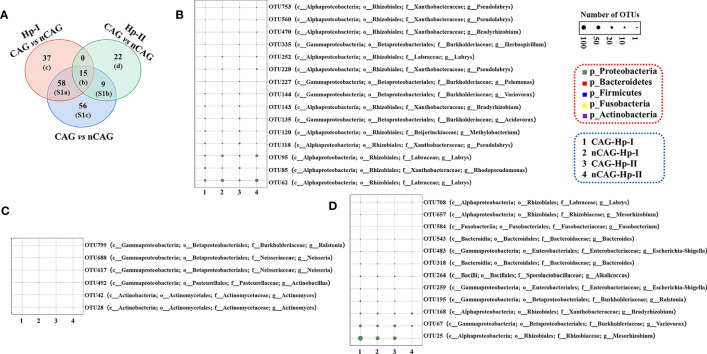
Different analysis of gastric microbiota in CAG patients with Hp infection. **(A)** Based on nCAG patients, Venn analysis of gastric different OTUs in CAG patients was performed among total population, Hp-I infected patients, and Hp-II infected patients. **(B)** The abundant OTUs was related to CAG risk. **(C)** The abundant OTUs was related to the risk of Hp-I induced CAG. **(D)** The abundant OTUs was related to the risk of Hp-II induced CAG.

On the one side, here observed the effect of CAG on gastric microbiota in the three respective groups (the total population, the Hp-I infected patients, and the Hp-II infected patients) (**Figure 3**). Venn intersection analysis obtained 197 differential OTUs between CAG and nCAG. Hp-I infected patients had much more differential OTUs than Hp-II infected patients, and 15 shared OTUs may be the different bacteria to distinguish CAG from nCAG (**Figure 3A**). Here presented the abundant status of 69 differential OTUs with a prevalence rate greater than 10%. Among them, fifteen sharing differential OTUs were considered to be CAG-related bacteria independent of Hp-infection, and they belonged to phylum Proteobacteria, including genus *Labrys*, *Rhodopseudomonas*, *Pseudolabrys*, *Methylobacterium*, *Acidovorax*, *Bradyrhizobium*, *Variovorax*, *Pelomonas*, and *Herbaspirillum* (**Figure 3B**). In Hp-I infected patients, 37 unique differential OTUs were regarded as CAG-related bacteria, among them, four abundant OTUs belonged to phylum Proteobacteria and Actinobacteria, including genus *Actinomyces*, *Neisseria*, *Actinobacillus* and *Ralstonia* (**Figure 3C**). In Hp-II infected patients, 22 unique differential OTUs were regarded as CAG-related bacteria, among them, 12 abundant OTUs belonged to the phylum Proteobacteria, Fusobacteria, Firmicutes and Bacteroidetes, including genus *Labrys*, *Mesorhizobium*, *Fusobacterium*, *Bacteroides*, *Escherichia-Shigella*, *Alkalicoccus*, *Ralstonia*, *Bradyrhizobium* and *Variovorax* (**Figure 3D**). In addition, the other shared OTUs in the Venn were considered as environment-related bacteria, all of which belonged to the phylum Proteobacteria (**Figure S1**). The results suggested that the gastric microbiota differ significantly during the progression from nCAG to CAG along with Hp-infection typing, which again confirmed that gastric non-*Helicobacter* bacteria play a certain role in the evolution of gastric mucosal inflammation and carcinoma, and Hp typing might be the key role in shaping the gastric microbiome.

On the other hand, here also explored the effect of Hp-infection typing on the gastric microbiota in CAG and nCAG patients, and observed 334 and 309 Hp-infection related OTUs in CAG and nCAG patients, respectively (**Figure S2**). Venn analysis showed that seven OTUs were associated with Hp-infection types, 28 OTUs were related to Hp-infection typing in CAG patients, and 86 OTUs linked with Hp-infection typing in nCAG patients (**Figure S2A**). Among the seven shared OTUs, Hp-I infected patients had a higher abundance of OTU1 (*Helicobacter pylori* 26695-1) than Hp-II infected patients, but had lower abundances of six non-*Helicobacter* OTUs (**Figure S2B**). Furthermore, OTU1 was a typical Hp-I strain with *CagA* and *VacA* genes, whose prevalence rate was 95.9% (185/193) in total population, suggesting that virulent Hp-infection inhibit the proliferation of non-*Helicobacter* bacteria in the stomach of gastritis patient. Compared with Hp-II infected patients, regardless of CAG (**Figure S2C**) or nCAG (**Figure S2D**), most of the differential OTUs were significantly decreased in the stomach of Hp-I infected patients. In the CAG patients, the significantly decreased OTUs were mainly oral dominant microbiota, such as genus *Neisseria*, *Staphylococcus*, *Haemophilus*. However, in the nCAG patients, the significantly reduced OTUs were mainly the gastric dominant microbiota, such as *Aliidiomarina*, *Reyranella*, *Halomonas*, *Pseudomonas*, and *Acidovorax*. The results suggest that virulent Hp infection inhibit the proliferation of normal gastric microbiota at the early stage of chronic gastritis, while inhibit the colonization of oral microbiota in the stomach at the late stage of chronic gastritis.

### 3.4 Hp infection altered symbiotic relationship of gastric microbiota in patients with chronic gastritis

**Figure 4:**
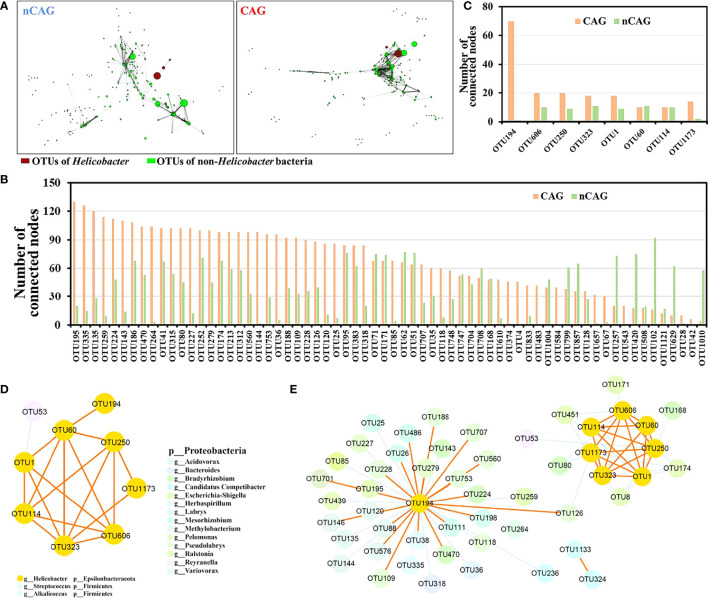
Effect of Hp-infection typing on the symbiotic network of gastric microbiota in patients with chronic gastritis. **(A)** Spearman correlation analysis was conducted to build the symbiotic networks at OTU level in CAG and nCAG patients, respectively. The red nodes referred to *Helicobacter* OTUs and the green nodes referred to non-*Helicobacter* OTUs, the size of node mean the relative abundance, the length of line mean absolute value of correlation coefficient (r). **(B)** Correlated number of 66 differential non-*Helicobacter* OTUs was extracted from the symbiotic network of gastric microbiota in CAG and nCAG patients. **(C)** Correlated number of eight *Helicobacter* OTUs was extracted from symbiotic network of gastric microbiota in CAG and nCAG patients. The symbiotic network was displayed between eight *Helicobacter* OTUs and non-*Helicobacter* OTUs in CAG **(D)** and nCAG patients **(E)**, respectively. The yellow solid line indicated positive correlation, while the blue dashed line indicated negative correlation.

Based on the previous literature (Yang et al., 2022), Spearman correlation analysis was performed at OUT level, and a significant correlation (*P* < 0.001) with absolute values of correlation coefficients |r| > 0.6 was selected to construct gastric symbiosis network in CAG and nCAG patients, respectively (**Figure 4**, **Table S2**). The gastric symbiosis network of nCAG patients included 290 nodes, and eight nodes belonging to *Helicobacter* genus significantly linked with sparse OTUs of non-*Helicobacter* bacteria. On the contrary, in the stomach of the CAG patients, the symbiosis network included 342 nodes, and the *Helicobacter* OTUs linked with numerous non-*Helicobacter* bacteria (**Figure 4A**).

Here focused on 127 differential OTUs in the network, the OTUs in the symbiotic network of CAG patients linking more than 30 nodes were defined as core OTUs, and 40 core OTUs were identified in the symbiotic network of CAG patients. Among the 40 core OTUs, ten OTUs (OTU195, OTU315, OTU374, OTU186, OTU312, OTU213, OTU41, OTU833, OTU174, OTU35) belonged to genus *Ralstonia*, nine OTUs (OTU753, OTU560, OTU109, OTU228, OTU188, OTU118 OTU126, OTU707, OTU748) belonged to genus *Pseudolabrys*, phylum Proteobacteria. Similarly, five core OTUs were identified in the symbiotic network of nCAG patients, and they were OTU629 (*Nesterenkonia*), OTU257 (*Herbaspirillum*), OTU1010 (*Halomonas*), OTU420 (*Cupriavidus*), and OTU102 (*Acidovorax*) (**Figure 4B**). The eight *Helicobacter* OTUs (OTU194, OTU606, OTU250, OTU323, OTU1, OTU60, OTU114, OTU1173) were extracted from the commensal network, and found that they had much more connections in the symbiotic network of CAG patients than that of nCAG patients (**Figure 4C**). Furthermore, in the stomach of nCAG patients, only OTU1 was significantly negatively associated with a non-*Helicobacter* OTU53 (*Reyranella*), and the symbiotic network was mainly composed of *Helicobacter* OTUs (**Figure 4D**). Meanwhile, in CAG patients, *Helicobacter* OTU194 and OTU1173 were associated with OTU126 (*Pseudolabrys*), and *Helicobacter* OTU194 linked with 35 non-*Helicobacter* OTUs including eight *Pseudolabs* OTUs, six *Ralstonia* OTUs, five *Bradyrhizobium* OTUs, five *Mesorhizobium* OTUs, and four *Variovorax* OTUs (**Figure 4E**). Overall, in the stomach of nCAG patients, *Helicobacter* bacteria might synergistically proliferate, and inhibit the proliferation of non-*Helicobacter* bacteria; in the stomach of CAG patients, both synergistic proliferation and antagonistic competition were observed between the *Helicobacter* bacteria and non-*Helicobacter* bacteria. The results provided new clues for probiotic application in preventing and treating Hp infection in patients with chronic gastritis.

### 3.5 Hp infection affected predictive function of gastric microbiota in patients with chronic gastritis

**Figure 5:**
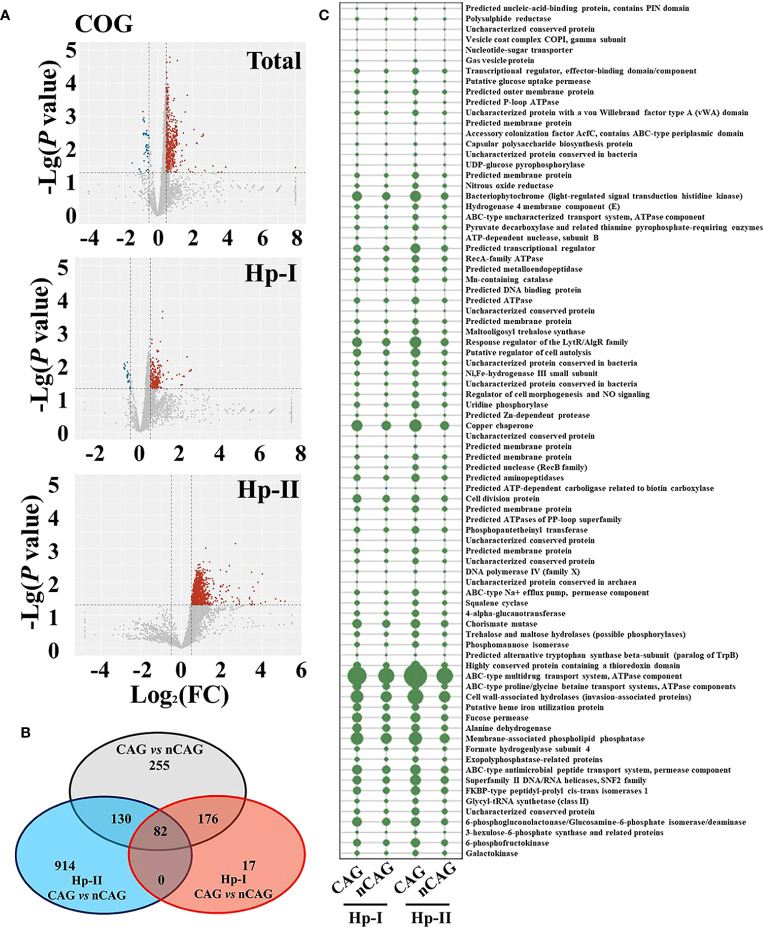
Effect of Hp-infection typing on the COG predictive function of gastric microbiota in patients with chronic gastritis. **(A)** Based on the nCAG patients, Volcano plots presented the different COG predictive functions of gastric microbiota in total population, Hp-I infected patients, and Hp-II infected patients, respectively. **(B)** Venn analysis was performed to screen the sharing and unique differential KEGG predictive functions. **(C)** The abundances of 82 sharing differential COG predictive functions were demonstrated in the four groups.

Based on the COG prediction function of gastric microbiota, here explored the potential signaling pathways in the development of CAG (**Figure 5**). With the criterion of Log_2_ (FC) > 0.5 or < - 0.5, and *P <*0.05, there were 605 up-regulated and 38 down-regulated COG predictive functions in CAG patients compared to nCAG patients. Among Hp-I infected patients, CAG patients had 251 up-regulated and 24 down-regulated COG predictive functions compared to nCAG patients; and in Hp-II infected patients, 1126 COG predictive functions were up-regulated in CAG patients compared to nCAG patients (**Figure 5A**). Venn analysis showed that 82 sharing differential COG prediction functions were related to the occurrence of CAG independent of Hp-infected type (**Figure 5B**). Among the 82 sharing functions, seven enhanced COG predictive functions included ATPase component, cell wall-associated hydrolases, membrane-associated phospholipid phosphatase, copper chaperone, response regulator of the LytR/AlgR family, bacteriophytochrome, and ABC-type Na^+^ efflux pump, permease component (**Figure 5C**). Since the seven functions links with microbial physiological metabolism, the gastric non-*Helicobacter* bacteria should also play certain role in the evolution of the Hp-induced nCAG to CAG, even to gastric carcinoma. For KEGG prediction function, there were 23 up-regulated and one down-regulated KEGG prediction functions. Twelve up-regulated and one down-regulated KEGG prediction functions were related to CAG with Hp-I infection, while 155 up-regulated KEGG prediction functions were related to CAG with Hp-II infection (**Figure S3A**). Venn analysis showed that only two sharing differential prediction functions linked with Hp-infection typing (**Figure S3B**), namely, sphingolipid metabolism and butirosin and neomycin biosynthesis were significantly enhanced in CAG patients (**Figure S3C**). These data provided new clues for understanding the development of Hp-infection inducing CAG.

### 3.6 Association analysis among gastric microbiota, living habits and clinical features in patients with chronic gastritis

Smoking and drinking are the two famous living habits for GC risk, and bile reflux and inflammatory activity are the two pathologic factors driving the progression of nCAG to CAG/GC (Poorolajal et al., 2020). Several studies proved that smoking and drinking could alter the gastrointestinal microbiota. Leite et al. (2022) found that smoking distinctly altered the duodenal microbiota, but quitters and non-smokers occupied similar duodenal microbiota, suggesting that living habits-induced microbial alteration is plastic and reversible. Bai et al. (2022) found that the exposure of cigarette smoking could increase the incidence of colorectal cancer in mice, accompanied with the absence of gut *Parabacteroides distasonis* and *Lactobacillus* spp., and the increased *Eggerthella lenta* was positively related to the fecal taurine deoxycholic acid (TDCA), which could in turn activate the intestinal epithelial MAPK/ERK pathway. Meanwhile, cigarette smoking could reduce the expressive levels of intestinal mucosal junction protein-3 and ZO-1, but increase the serum LPS level, leading to intestinal barrier damage and colorectal cancer. Zhao et al. (2022) found that the occurrence of alcoholic fatty liver was associated with altered gut microbiota, while Nie et al. (2022) found that the long-chain saturated fatty acids (palmitic acid, stearic acid) in Jinhua ham could prevent chronic alcohol-associated liver damage (ALD), and the preventive effect was associated with increased gut *Akkermansia muciniphila* and *Lactobacillus*. Chen et al. (2022) found that *Bacteroides xylanisolvens* could degrade the cumulative nicotine in the intestine, and prevent the development of nonalcoholic fatty liver disease (NAFLD). However, the relationship between smoking/drinking and gastric microbiota is still unclear in the occurrence of CAG.

**Figure 6:**
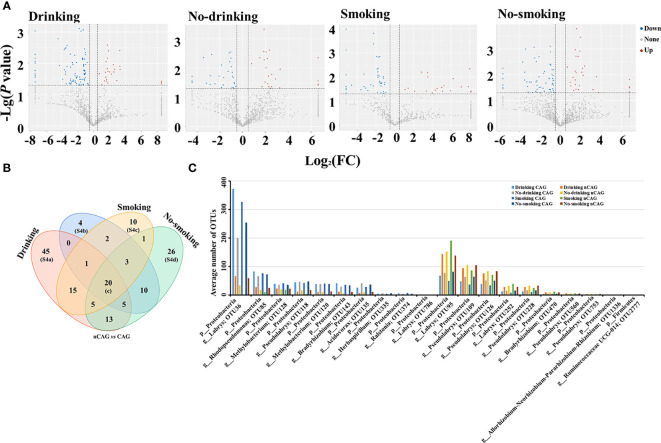
Effect of living habits on gastric microbiota in patients with chronic gastritis. **(A)** Based on the nCAG patients, Volcano plots presented the different gastric OTUs in drinking patients, no-drinking patients, smoking patients, and no-smoking patients. **(B)** Venn analysis was performed to screen the sharing and unique different OTUs. **(C)** The 20 shared OTUs were presented in patients with certain living habit.

Thus, this study investigated relationship between smoking/drinking and gastric microbiota in CAG patients (**Figure 6**). Among drinkers, the CAG patients occupied 26 significantly up-regulated and 94 down-regulated bacterial OTUs compared to nCAG patients; among non-drinkers, there were 24 significantly up-regulated and 33 down-regulated bacterial OTUs in CAG patients compared to nCAG patients. Similarly, compared to smoking nCAG patients, 53 down-regulated and 20 up-regulated bacterial OTUs were observed in the smoking CAG patients; among the non-smokers, 85 down-regulated and 29 up-regulated bacterial OTUs were found in CAG patients compared to nCAG patients (**Figure 6A**). Venn analysis showed that 20 sharing bacterial OTUs might be related to CAG development. The drinking patients had 45 unique OTUs, while the non-drinking patients had four unique OTUs; the smoking patients had ten unique OTUs, while the non-smoking patients had 26 unique OTUs (**Figure 6B**). Here demonstrated the dominant OTUs in the stomach of chronic gastritis patients with different lifestyle status. Among the 20 sharing OTUs, the ten up-regulated OTUs belonged to genus *Labrys*, *Rhodopseudomonas* and *Methylobacterium*, while the ten down-regulated OTUs mainly belonged to *Labrys* and *Pseudolabrys*, suggesting that gastric *Labrys* play a key role in the development of CAG (**Figure 6C**). In drinking CAG patients, among the 45 unique OTUs, most of them were significant down-regulated, and belonged to genus *Bradyrhizobium*, *Aliidiomarina* and *Halomonas* (**Figure S4A**). In non-drinking CAG patients, among the four unique OTUs, the increasing gastric OTU341 belonged to phylum Actinobacteria (**Figure S4B**). In smoking CAG patients, among the ten unique OTUs, increasing OTU439 (genus *Ralstonia*) and decreased OTU1067 (genus *Labrys*) were observed (**Figure S4C**). Among the 26 unique OTUs in the non-smoking patients, CAG patients occupied an elevated OTU323 (genus *Helicobacter*) with decreasing OTU21 (genus *Prevotella 7*) and OTU704 (genus *Acidovorax*) (**Figure S4D**). Compared to nCAG patients, most non-*Helicobacter* bacteria were significantly decreased in CAG patients, which was consistent with the decreased richness of gastric microbiota in CAG patients, suggesting that Hp-infection may inhibit the proliferation of non-*Helicobacter* bacteria in the stomach of CAG patients.

Moreover, the present study explored the effects of smoking, alcohol consumption, bile reflux, and inflammatory activity on the gastric microbiota in CAG and nCAG patients, respectively (**Figure S5**). Comparative analysis was conducted between smoking and non-smoking individuals, only one different OUT was observed in the stomach of both nCAG and CAG patients (**Figure S5A**). Similarly, comparative analysis was conducted between drinking and non-drinking individuals, even no different OUT was observed in the stomach of both nCAG and CAG patients (**Figure S5B**). These results suggested that gastritis development stage, but not smoking/drinking, play a key role in shaping the gastric microbiota. Compared to the patients without inflammatory activity, there were 12 down-regulated and 24 up-regulated OTUs in the stomach of nCAG patients, and 56 down-regulated and six up-regulated OTUs in the stomach of CAG patients. Three sharing differential OTUs (OTU1, OTU60, OTU194) belonged to genus *Helicobacter*, and OTU1969 belonged to genus *Streptococcus* (**Figure S5C**), suggesting that *Helicobacter* bacteria are the key bacteria inducing gastric inflammatory activity. Due to the low prevalence rate of bile reflux in nCAG patients, here just observed the effect of bile reflux on gastric microbiota in CAG patients. Compared with CAG patients without bile reflux, 307 up-regulated OTUs were observed in the stomach of CAG patients with bile reflux, and most of the up-regulated OTUs were oral dominant bacteria, such as *Veillonella*, *Prevotella 7* and *Rothia* (**Figure S5D**). These results suggested that bile reflux could promote the colonization of oral microbiota in the gastric mucosa of CAG patients.

**Figure 7:**
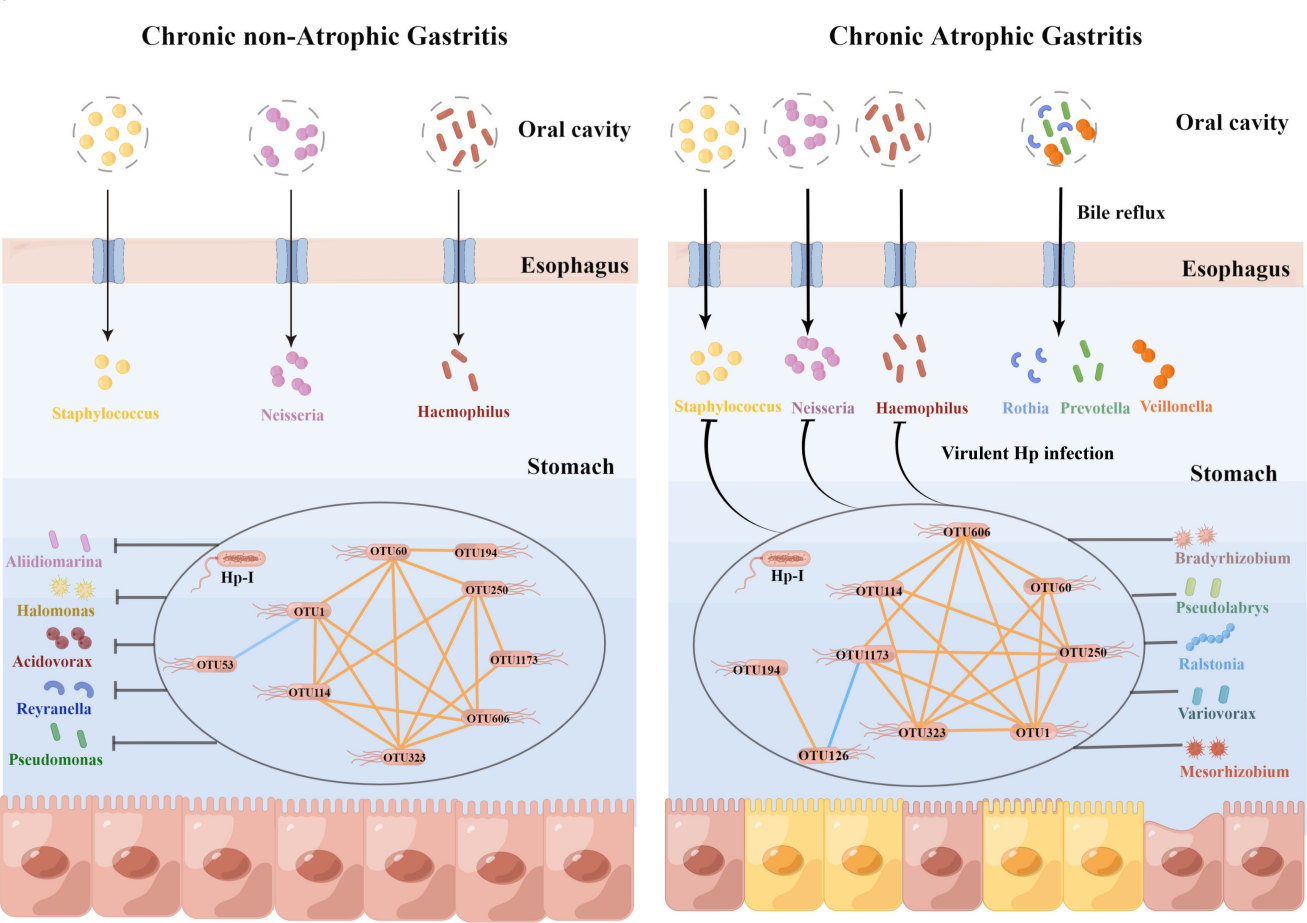
Hp-infection typing exerted deeply impact on the gastric microbiota in the chronic gastritis patients. This figure was drawn by Figdraw (https://www.figdraw.com/).

In summary, several interrelated *Helicobacter* bacteria were observed in the stomach of gastritis patients with Hp-infection. At the phase of nCAG, *Helicobacter* bacteria were almost unrelated to the non*-Helicobacter* bacteria, and virulent Hp-I infection decreased gastric dominant bacteria including *Aliidiomarina*, *Reyranella*, *Halomonas*, *Pseudomonas* and *Acidovorax*. In case of CAG development, bile reflux increased oral microbiota (*Rothia*, *Prevotella*, *Veillonella*) to colonize in the gastric mucosa, and in the virulent Hp-infected patients, *Helicobacter* bacteria linked closely with non*-Helicobacter* bacteria (*Bradyrhizobium*, *Psedolabrys*, *Ralstonia*, *Mesorhizobium* and *Variovorax*), and inhibited oral microbiota from colonizing in the gastric mucosa (**Figure 7**). Thus, the results bring new understanding of etiology in the development of chronic gastritis.

## 4 Discussion

GC is one of the major cancers in China and all over the world. China has developed the government-funded national screening programs for GC from 2008, and effectively decreased the incidence and mortality (Fan et al., 2021). Hp-infection leads to chronic persistent inflammatory lesions of gastric mucosa, and has been recognized as a classic etiology for GC risk. Large-scale, national, family-based epidemiological study showed that family Hp-infection is still up to 71.2% in China (Zhou et al., 2023). However, only 3% of Hp-infected individuals develop to GC, suggesting that gastric non-Hp microbiota involve in gastric carcinogenesis. Lofgren et al. (2011) reported that gut microbiota could accelerate gastric carcinogenesis, while antibiotic could delay the development of GC in Hp-infection or uninfected insulin-gastrin mice. More and more evidences showed that gastric microbiota involved in the cascade response of gastric carcinogenesis. For example, Coker et al. (2018) analyzed the gastric microbiota in four stages of gastric carcinogenesis: superficial gastritis (SG), atrophic gastritis (AG), intestinal metaplasia (IM) and GC, the results showed that gastric *Peptostreptococcus stomatis*, *Streptococcus anginosus*, *Parvimonas micra*, *Slackia exigua* and *Dialister pneumosintes* were enriched in GC group, especially the Hp-negative patients with SG and IM had more closely symbiotic networks of gastric microbiota; Liu et al. (2019) designed a cohort study and found that GC patients had decreased diversity of gastric microbiota and simpler symbiotic relationship, several gastric bacteria (*Hp*, *Prevotella copri* and *Bacteroides uniformis*) significantly decreased, but the common oral bacteria (*Prevotella melaninogenica*, *S. anginosus*, *Propionibacterium acnes*) significantly increased in gastric cancer, and higher load of Hp-infection linked with the distinct gastric microbiota. Sun et al. (2022) presented the alteration of gastric microbiota in Hp-negative population from SC to GC, and found that the gastric microbial diversity continued to decline, with more complex symbiotic networks of gastric microbiota were observed in patients with IM and heterogeneous hyperplasia. These results suggested that Hp infection obviously altered gastric microbiota, but the effect of Hp-infection typing on gastric microbiota is still unclear.

The present study showed that the most abundant phylum was Proteobacteria, which included the dominant bacteria genus *Helicobacter*, and the no-*Helicobacter* bacteria were *Ralstonia*, *Pseudonocardia*, *Mesorhizobium*, *Bradyrhizobium* and *Labrys*, etc. Sun et al. (2022) also observed the dominant *Ralstonia* and *Rhodococcus* in the stomach of Hp-negative IM and Dys patients. The *Ralstonia* was Gram-negative, widely observed in water supply systems, and able to adapt to low nutrient environments. Up to now, six *Ralstonia* species have been identified: *R. insidiosa*, *R. mannitolilytica*, *R. pickettii*, *R. pseudosolanacearum, R. solanacearum*, and *R. syzygii*. Three species (*R. pickettii*, *R. insidiosa* and *R. mannitolilytica*) could cause opportunistic infections, such as osteomyelitis and meningitis; the other three species (*R. pseudosolanacearum*, *R. solanacearum*, and *R. syzygii*) were important phytopathogenic bacteria (Ryan and Adley, 2014). *R. mannitolilytica* was often found in cystic fibrosis, Fluit et al. (2021) analyzed the whole genome of 18 strains of *Ralstonia*, and all strains occupied resistance genes encoding β-lactamases of OXA-22 and OXA-60 family, but no potential virulence factors were observed. Since the resistant Hp has become the greatest challenge in the global prevention and treatment of Hp infection (Flores-Treviño et al., 2018), it may be boldly speculated that *Ralstonia* serve as the donor bacteria of potential resistance genes for Hp resistance variation.

Several studies have shown that the dominant non-*Helicobacter* bacteria in gastric mucosa are much more complex and diverse. For example, Zhang et al. (2021) found that the dominant gastric mucosal microbiota in 47 patients with CSG to GC included *Streptococcus*, *Bacillus* and *Anaerobacillus*, and the abundance of *Helicobacter* was significantly higher in patients with cardia GC than that in patients with non-cardia GC. Liu et al. (2019) observed the characteristics of gastric microbiota in 276 patients with GC, and found that the cancerous, paracancerous and normal tissues occupied similar dominant bacteria, among which *Helicobacter*, *Halomonas*, and *Shewanella* were enriched in paracancerous tissues, while *Streptococcus*, *Selenomonas*, *Fusobacterium*, *Propionibacterium*, and *Corynebacterium* were enriched in cancerous tissues. Liatsos et al. (2022) proposed that Hp infection could lead to gastric mucosal dysbiosis and inflammatory carcinogenesis, mainly manifested as decreasing colonization of non-*Helicobacter* bacteria in the stomach and lower gastric microbial diversity. Similar findings were found in this study, where the rate of Hp-I infection was significantly higher in CAG patients than in nCAG patients, and the gastric microbial richness (Observed OTUs, Chao) was significantly decreased in CAG patients compared with nCAG patients. Moreover, in CAG patients, Hp-I infected patients had lower gastric microbial diversity (Shannon index) than Hp-II infected patients. Compared to Hp-II infected patients, most gastric OTUs were significantly decreased in Hp-I infected patients. Surprisingly, in nCAG patients, the decreased OTUs mainly belonged to genus *Aliidiomarina*, *Reyranella*, *Halomonas*, *Pseudomonas* and *Acidovorax*; in CAG patients, the decreased OTUs were mainly oral dominant bacteria including *Neisseria*, *Staphylococcus* and *Haemophilus*, which was consistent with the previous literature (Sun et al., 2022) that dominant oral bacteria (*Streptococcaceae* and *Prevotellaceae*) continued to decrease in the stomach of CAG and dysplasia patients. Furthermore, this study found that CAG patients with bile reflux had more abundant oral bacteria (*Veillonella*, *Prevotella 7* and *Rothia*) in the gastric mucosa than CAG patients without bile reflux, suggesting that oral microbiota were more likely to colonize and proliferate in the stomach of CAG patients with bile reflux. It can be seen that virulent Hp infection could reduce the diversity of gastric microbiota, decrease the abundance of normal gastric microbiota in nCAG patients, while decrease the abundance of oral dominant microbiota in the stomach of CAG patients. Recently, probiotic-assisted therapy has become a new strategy for the eradication of resistant Hp or combined with triple therapy (Goderska et al., 2018), and the present data provided a new scientific basis for intervention of microbiota preparation in ameliorating gastric microbiota to prevent and treat Hp infection.

Finally, Spearman correlation analysis was conducted to observe the symbiotic relationship between *Helicobacter* and non-*Helicobacter* bacteria. In nCAG patients, an internal positive symbiotic network among the eight *Helicobacter* OTUs was observed in the gastric microbiota, but only non-*Helicobacter* OTU53 (genus *Reyranella*) was significantly negatively correlated to OTU1 (*Helicobacter pylori 26695-1*). On the contrary, in CAG patients, not only significant positive symbiotic network was observed within the eight *Helicobacter* OTUs, but also the eight *Helicobacter* OTUs correlated with dozens of non-*Helicobacter* OTUs, which belonged to *Pseudolabrys*, *Ralstonia*, *Bradyrhizobium*, *Mesorhizobium*, and *Variovorax*. The results suggested that *Helicobacter* bacteria evolved a close symbiotic network in the stomach of gastritis patients, and the symbiotic network linked closely with the non- *Helicobacter* bacteria when CAG occurred. Thus, Hp eradication inevitably led to gastric dysbiosis in CAG patients, accompanying by dyspeptic symptoms such as nausea, vomiting and diarrhea (Khoshnood et al., 2014). Recent clinical evidence showed that the clinical outcome of Hp-infection was influenced by many factors including climate, geography, host immunity, gut microbiota, nutritional status and medications (Alexander et al., 2021). Therefore, Hp-infection typing and gastric symbiotic status should also be important factors affecting the clinical prognosis of Hp infection, which provides new clues for the clinical management of Hp infection in patients with chronic gastritis.

In summary, this study explored the effect of Hp-infection typing on the characteristics of gastric microbiota in the gastritis patients, and main findings included three points: 1) virulent Hp-infection obviously reduced the richness of gastric microbiota; 2) virulent Hp infection inhibited the dominant gastric microbiota in patients with nCAG, such as *Aliidiomarina*, *Reyranella*, *Halomonas*, *Pseudomonas* and *Acidovorax*; but in CAG patients, virulent Hp infection inhibited the oral common bacteria colonizing in the gastric mucosa, such as *Neisseria*, *Staphylococcus* and *Haemophilus*. 3) *Helicobacter* bacteria correlated closely in the stomach of gastritis patients with Hp-infection, while CAG development greatly promoted the correlation between *Helicobacter* bacteria and non-*Helicobacter* bacteria. Of course, there were also some limitations of this study: 1) the study population was limited to a single region and need to be validated in more regional populations; 2) the specificity of the RUT was susceptible to the interference of urease-positive bacteria, and more precise typing of Hp-infection required PCR verification; 3) the Hp-infection typing based on serum Hp antibody could not distinguish between current infection and previous infection. Anyway, this study provides a new insight into understanding the effect of Hp-infection on gastric microbiota in patients with chronic gastritis, and lays the foundation for microbial intervention to prevent and treat Hp infection.

## Data availability statement

The raw data of gastric 16SrDNA sequences presented in the study are deposited in the NCBI Sequence Read Archive (SRA) database, accession number SRP437805.

## Ethics statement

The study was approved by the Ethics Committee of People’s Hospital of Yangzhong City (No.2018-039) and the patients gave informed consent. The patients/participants provided their written informed consent to participate in this study.

## Author contributions

QZ and JZ designed the study. ZH, LeX, and JZ wrote the manuscript. ZH and BL managed the clinical sampling. LeX, JhZ, LX, JW, ZW, and QZ analyzed the data. ZH and LX contributed equally for this manuscript preparation. LeX and JZ revised the manuscript. All authors reviewed the manuscript. All authors contributed to the article and approved the submitted version.

## Supplementary material

Supplementary Figure 1 The abundances of the CAG-related OTUs in the four groups.

Supplementary Figure 2 Effect of Hp-infection typing on gastric microbiota in patients with chronic gastritis. **(A)** Differential analysis and Venn analysis were conducted between Hp-I and Hp-II infected patients in the CAG patients and nCAG patients, respectively. **(B)** The abundances of seven sharing OTUs were presented in four groups, respectively. **(C)** In CAG patients, the abundances of the 28 unique OTUs were presented in Hp-I and Hp-II infected individuals. **(D)** In nCAG patients, 25 abundant OTUs of the 86 unique OTUs were presented in Hp-I and Hp-II infected individuals.

Supplementary Figure 3 Effect of Hp-infection typing on the KEGG predictive function of gastric microbiota in patients with chronic gastritis. **(A)** Based on the nCAG patients, Volcano plots presented the different KEGG predictive functions of gastric microbiota in total population, Hp-I infected patients, and Hp-II infected patients, respectively. **(B)** Venn analysis was performed to screen the sharing and unique different KEGG predictive functions. **(C)** The abundances of two sharing different KEGG predictive functions were demonstrated in the four groups.

Supplementary Figure 4 The unique CAG-related bacteria in the stomach of patients with certain lifestyle status. Based on the nCAG patients, the unique different bacteria were presented in the patients with drinking **(A)**, non-drinking **(B)**, smoking **(C)**, and non-smoking **(D)**.

Supplementary Figure 5 Effect of clinical characteristics and living habits on gastric microbiota in patients with chronic gastritis. **(A)** Based on no-smoking individuals, different analysis and Venn analysis were conducted to screen the smoking-related bacteria in the CAG and nCAG patients, respectively. **(B)** Based on no-drinking individuals, different analysis and Venn analysis were conducted to screen the drinking-related bacteria in the CAG and nCAG patients, respectively. **(C)** Based on individuals with gastric inactive inflammation, different analysis and Venn analysis were conducted to screen the inflammation-related bacteria in the CAG and nCAG patients, respectively. **(D)** Based on individuals without bile reflex in CAG patients, different analysis was conducted to screen the bile reflex-related bacteria.

Supplementary Table 1 The common characteristics of the patients with chronic gastritis.

Supplementary Table 2 The symbiotic network of gastric microbiota in nCAG and CAG patients.
