# Supplementary figures and images for: Helicobacter pylori infection altered gastric microbiota in patients with chronic gastritis
Source: Front Cell Infect Microbiol. 2023 Aug 17;13:1221433. doi: 10.3389/fcimb.2023.1221433 (PMC10470091; doi:10.3389/fcimb.2023.1221433)

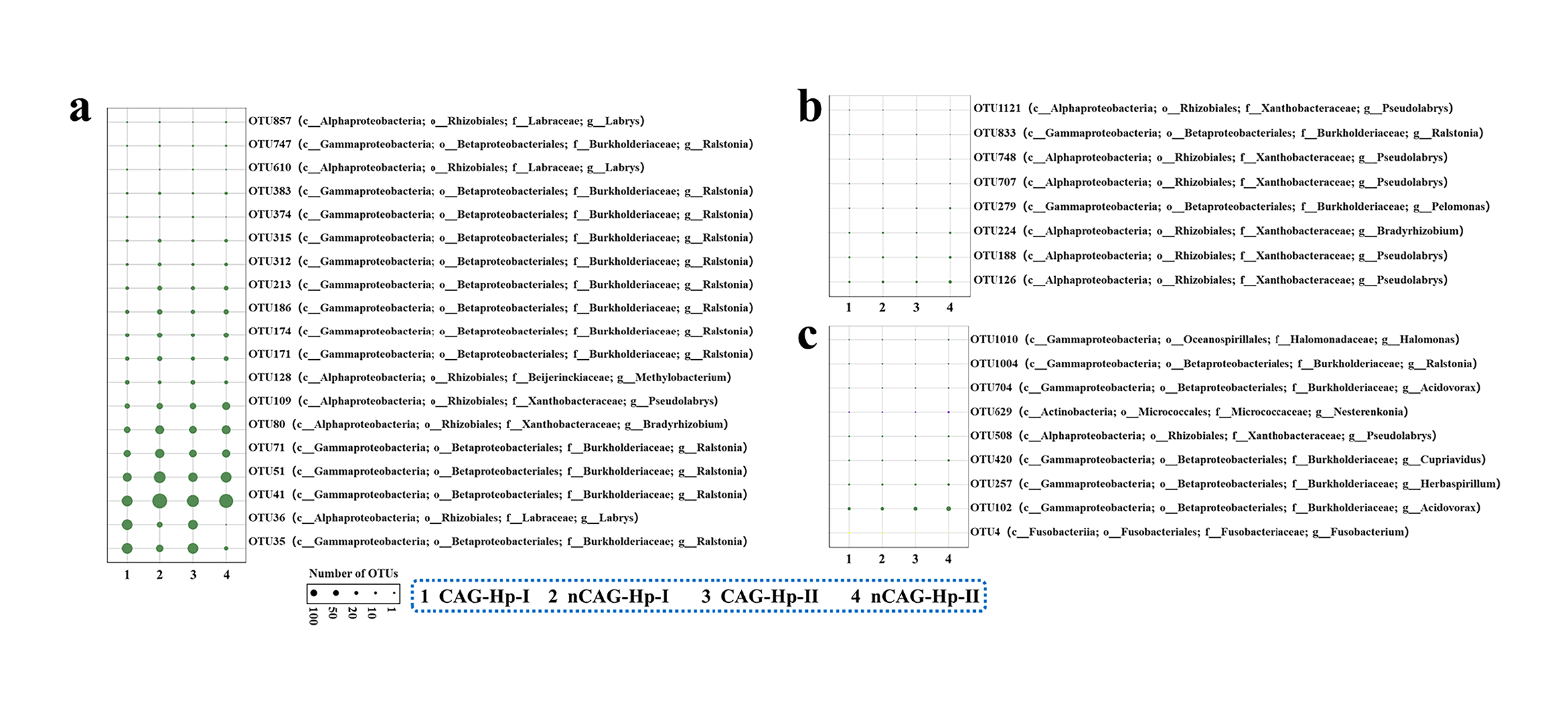

Supplement: Supplementary Figure 1 — The abundances of the CAG-related OTUs in the four groups. [file Image_1.tif]

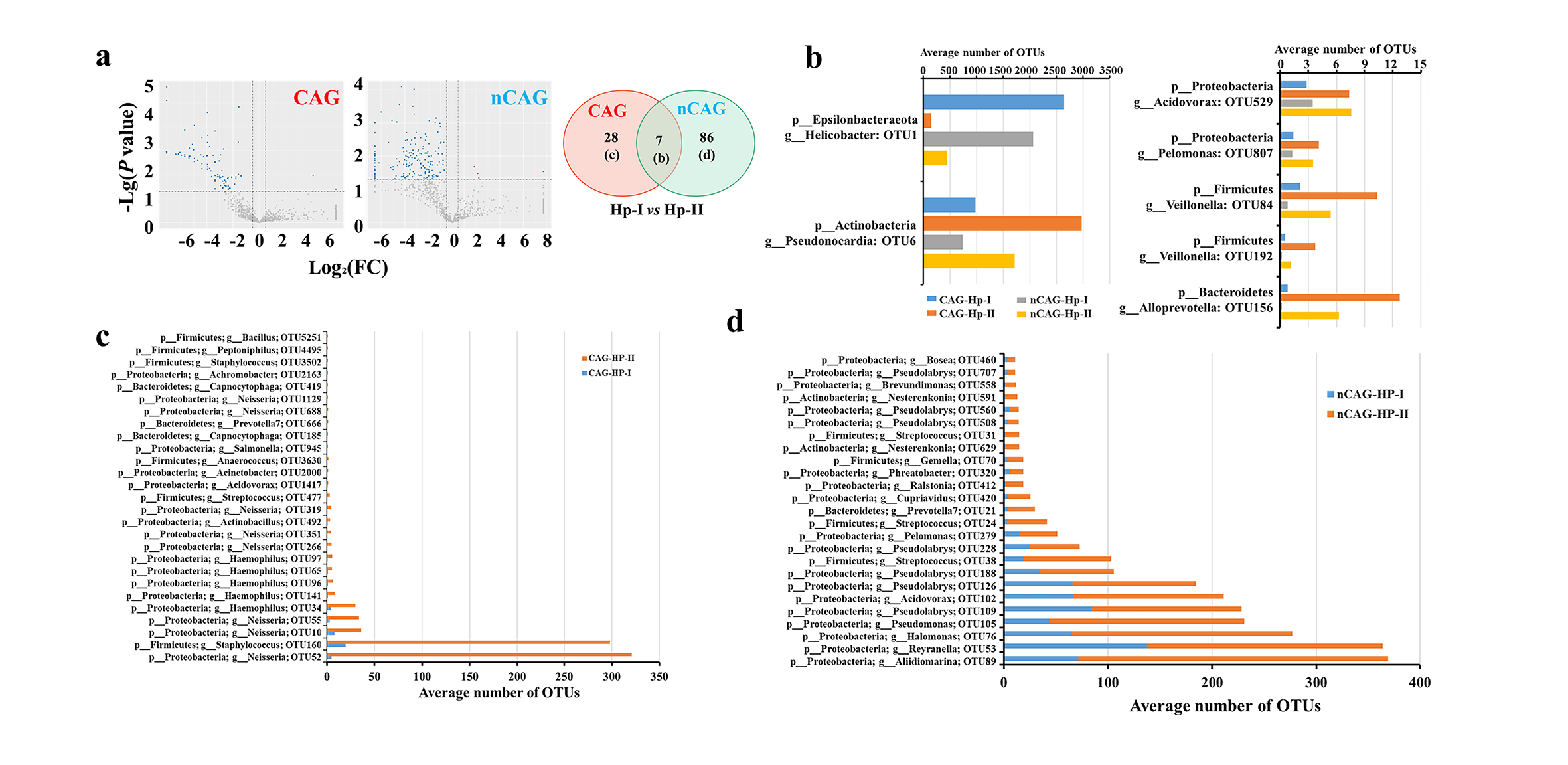

Supplement: Supplementary Figure 2 — Effect of Hp-infection typing on gastric microbiota in patients with chronic gastritis. (A) Differential analysis and Venn analysis were conducted between Hp-I and Hp-II infected patients in the CAG patients and nCAG patients, respectively. (B) The abundances of seven sharing OTUs were presented in four groups, respectively. (C) In CAG patients, the abundances of the 28 unique OTUs were presented in Hp-I and Hp-II infected individuals. (D) In nCAG patients, 25 abundant OTUs of the 86 unique OTUs were presented in Hp-I and Hp-II infected individuals. [file Image_2.tif]

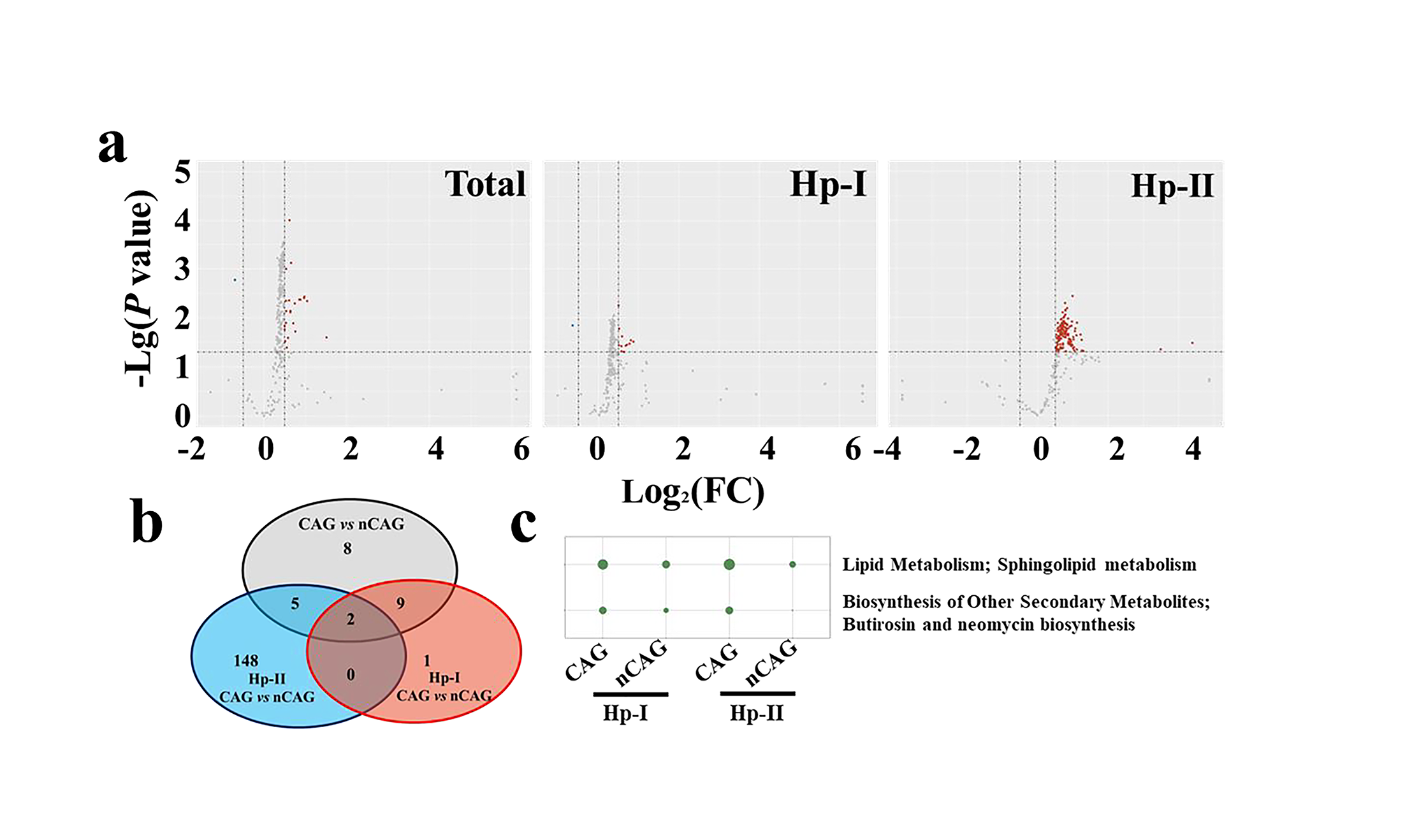

Supplement: Supplementary Figure 3 — Effect of Hp-infection typing on the KEGG predictive function of gastric microbiota in patients with chronic gastritis. (A) Based on the nCAG patients, Volcano plots presented the different KEGG predictive functions of gastric microbiota in total population, Hp-I infected patients, and Hp-II infected patients, respectively. (B) Venn analysis was performed to screen the sharing and unique different KEGG predictive functions. (C) The abundances of two sharing different KEGG predictive functions were demonstrated in the four groups. [file Image_3.tif]

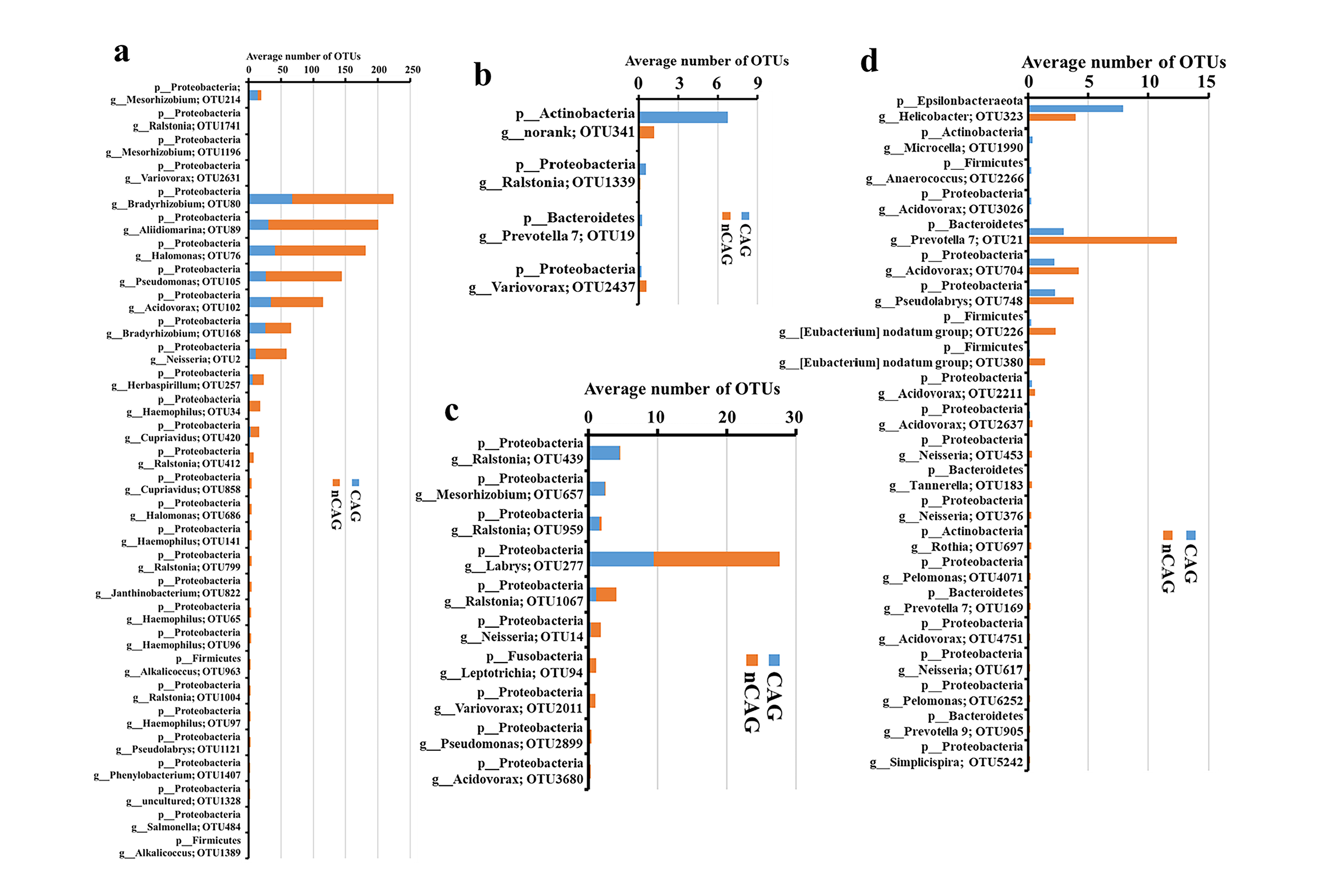

Supplement: Supplementary Figure 4 — The unique CAG-related bacteria in the stomach of patients with certain lifestyle status. Based on the nCAG patients, the unique different bacteria were presented in the patients with drinking (A), non-drinking (B), smoking (C), and non-smoking (D). [file Image_4.tif]

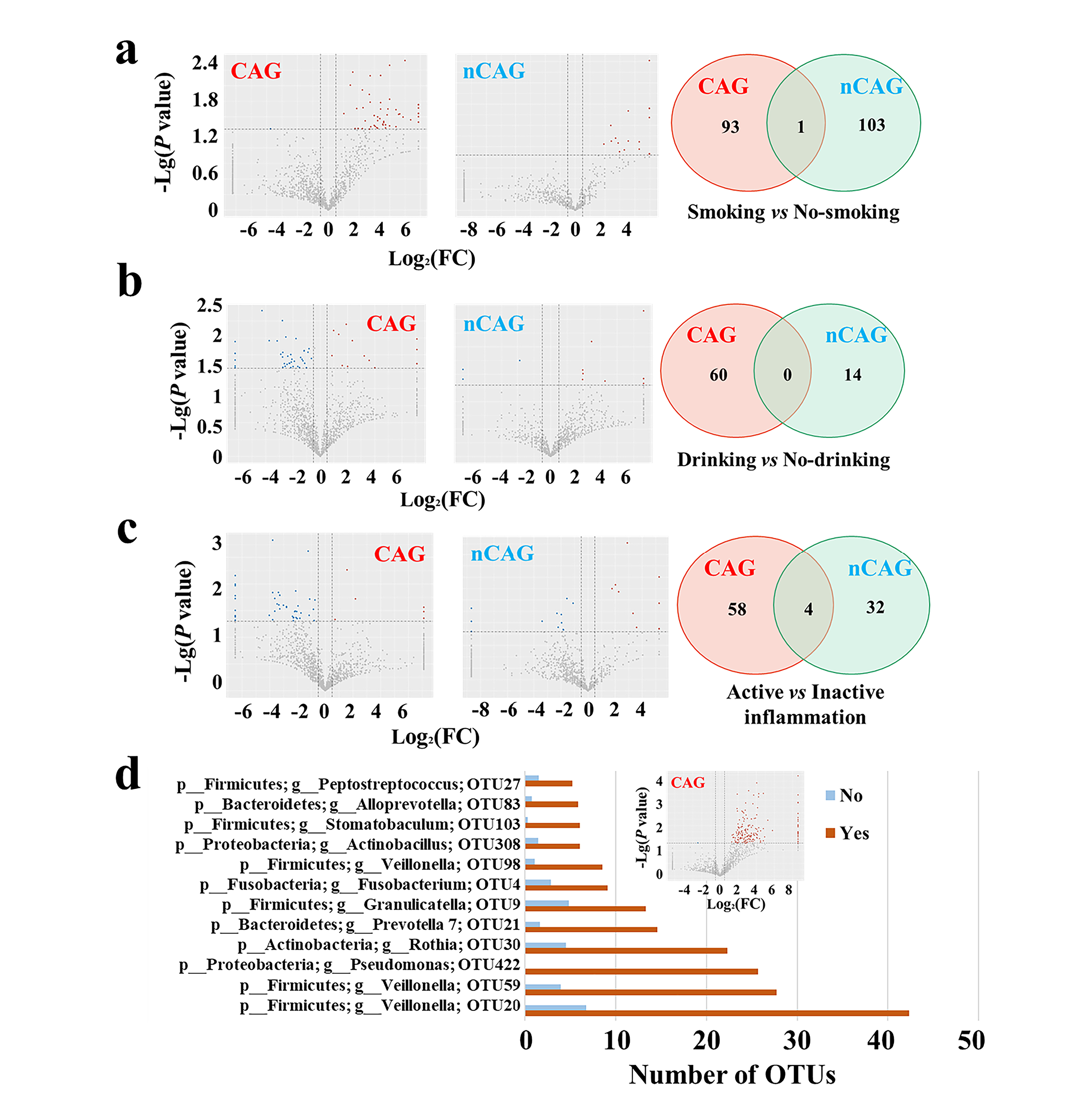

Supplement: Supplementary Figure 5 — Effect of clinical characteristics and living habits on gastric microbiota in patients with chronic gastritis. (A) Based on no-smoking individuals, different analysis and Venn analysis were conducted to screen the smoking-related bacteria in the CAG and nCAG patients, respectively. (B) Based on no-drinking individuals, different analysis and Venn analysis were conducted to screen the drinking-related bacteria in the CAG and nCAG patients, respectively. (C) Based on individuals with gastric inactive inflammation, different analysis and Venn analysis were conducted to screen the inflammation-related bacteria in the CAG and nCAG patients, respectively. (D) Based on individuals without bile reflex in CAG patients, different analysis was conducted to screen the bile reflex-related bacteria. [file Image_5.tif]
