# Supplementary material for: Helicobacter pylori infection altered gastric microbiota in patients with chronic gastritis
Source: Front Cell Infect Microbiol. 2023 Aug 17;13:1221433. doi: 10.3389/fcimb.2023.1221433 (PMC10470091; doi:10.3389/fcimb.2023.1221433)
Supplement: Supplementary Table 1 — The common characteristics of the patients with chronic gastritis. [file Table_1.docx]

**Table S1.** The common characteristics of the patients with chronic gastritis

|  |  | nCAG (n=69) | CAG (n=124) | *P* |
| --- | --- | --- | --- | --- |
| Age |  | 55.7±6.9 | 58.4±7.2 | 0.014* |
| BMI (kg/m^2^) |  | 24.1±3.7 | 23.9±3.0 | 0.817* |
| PG-I/PG-II |  | 4.6±3.6 | 3.9±2.2 | 0.113* |
| Gender | Male | 30(43.5%) | 57(46.0%) | 0.739 |
|  | Female | 39(56.5%) | 67(54.0%) |  |
| Type of Hp | Hp-I | 49(71.0%) | 113(91.1%) | < 0.001 |
|  | Hp-II | 20(29.0%) | 11(8.9%) |  |
| CagA-IgG | Negative | 21(30.4%) | 14(11.3%) | 0.001 |
|  | Positive | 48(69.6%) | 110(88.7%) |  |
| VacA-IgG | Negative | 34(49.3%) | 29(23.4%) | < 0.001 |
|  | Positive | 35(50.7%) | 95(76.6%) |  |
| Active inflammation | No | 46(66.7%) | 52(41.9%) | 0.001 |
|  | Yes | 23(33.3%) | 72(58.1%) |  |
| Bile reflux | No | 67(97.1%) | 90(72.6%) | 0.006 |
|  | Yes | 2(2.9%) | 33(26.6%) |  |
| Smoking | No | 55(79.7%) | 90(72.6%) | 0.272 |
|  | Yes | 14(20.3%) | 34(27.4%) |  |
| Drinking | No | 40(58.0%) | 71(57.3%) | 0.923 |
|  | Yes | 29(42.0%) | 53(42.7%) |  |
| Eating on time | Often | 59(85.5%) | 106(85.5%) | 0.721 |
|  | Sometime | 9(13.0%) | 14(11.3%) |  |
|  | Seldom | 1(1.4%) | 4(3.2%) |  |
| Eating speed | <10 min | 18(26.1%) | 16(12.9%) | 0.013 |
|  | 10~20 min | 43(62.3%) | 76(61.3%) |  |
|  | >20 min | 8(11.6%) | 32(25.8%) |  |
| Salt preference | Heavy | 21(30.4%) | 31(25.0%) | 0.006 |
|  | Moderate | 36(52.2%) | 44(35.5%) |  |
|  | Weak | 12(17.4%) | 49(39.5%) |  |
| Eating fried food | Never | 15(21.7%) | 35(28.2%) | 0.450 |
|  | Seldom | 54(78.3%) | 88(71.0%) |  |
|  | Often | 0(0.0%) | 1(0.8%) |  |
| Eating fresh fruits | Never | 5(7.2%) | 9(7.3%) | 0.455 |
|  | Seldom | 41(59.4%) | 84(67.7%) |  |
|  | Often | 19(27.5%) | 22(17.7%) |  |
|  | Daily | 4(5.8%) | 9(7.3%) |  |
| Eating pickled food | Never | 8(11.6%) | 7(5.6%) | 0.276 |
|  | Seldom | 42(60.9%) | 81(65.3%) |  |
|  | Often | 11(15.9%) | 27(21.8%) |  |
|  | Daily | 8(11.6%) | 9(7.3%) |  |

The asterisks refer to Student’s *t*-test, and the others refer to Chi-square test.
